# Stereotactic radiotherapy of brain metastases: clinical impact of three-dimensional SPACE imaging for 3T-MRI-based treatment planning

**DOI:** 10.1007/s00066-022-01996-1

**Published:** 2022-08-17

**Authors:** Thomas Welzel, Rami A. El Shafie, Bastian v. Nettelbladt, Denise Bernhardt, Stefan Rieken, Jürgen Debus

**Affiliations:** 1 Department of Radiation Oncology, Heidelberg University Hospital, Im Neuenheimer Feld 400, 69120 Heidelberg, Germany; 2 Heidelberg Institute of Radiation Oncology (HIRO), Heidelberg, Germany; 3 National Center for Tumor diseases (NCT), Heidelberg, Germany; 4 German Cancer Consortium (DKTK), Partner site Heidelberg, German Cancer Research Center (DKFZ), Heidelberg, Germany; 5 Department of Radiation Oncology, University Medical Center Göttingen, Robert-Koch-Str. 40, 37075 Göttingen, Germany; 6 Department of Radiation Oncology, Klinikum rechts der Isar, Technical University Munich, Ismaninger Str. 22, 81675 Munich, Germany

**Keywords:** Brain metastases, CyberKnife, Stereotactic radiosurgery, Variable flip angle, Magnetic resonance imaging

## Abstract

**Purpose:**

For planning CyberKnife stereotactic radiosurgery (CK SRS) of brain metastases (BM), it is essential to precisely determine the exact number and location of BM in MRI. Recent MR studies suggest the superiority of contrast-enhanced 3D fast spin echo SPACE (sampling perfection with application-optimized contrast by using different flip angle evolutions) images over 3D gradient echo (GE) T1-weighted MPRAGE (magnetization-prepared rapid gradient echo) images for detecting small BM. The aim of this study is to test the usability of the SPACE sequence for MRI-based radiation treatment planning and its impact on changing treatment.

**Methods:**

For MRI-based radiation treatment planning using 3T MRI in 199 patients with cerebral oligometastases, we compared the detectability of BM in post-gadolinium SPACE images, post-gadolinium MPRAGE images, and post-gadolinium late-phase MPRAGE images.

**Results:**

When SPACE images were used for MRI-based radiation treatment planning, 29.8% and 16.9% more BM, respectively, were detected and included in treatment planning than in the post-gadolinium MPRAGE images and the post-gadolinium late-phase MPRAGE images (post-gadolinium MPRAGE imaging: n_total_ = 681, mean ± SD 3.4 ± 4.2; post-gadolinium SPACE imaging: n_total_ = 884, mean ± SD 4.4 ± 6.0; post-gadolinium late-phase MPRAGE imaging: n_total_ = 796, mean ± SD 4.0 ± 5.3; *P*_post-gadolinium SPACE imaging versus post-gadolinium MPRAGE imaging_ < 0.0001, *P*_post-gadolinium SPACE imaging versus post-gadolinium late-phase MPRAGE imaging_< 0.0001).

**Conclusion:**

For 3T MRI-based treatment planning of stereotactic radiosurgery of BM, we recommend the use of post-gadolinium SPACE imaging rather than post-gadolinium MPRAGE imaging.

## Introduction

Magnetic resonance imaging (MRI) is a safe and effective diagnostic examination technique for radiation treatment planning for cerebral metastases. The decision on the optimal radiotherapy for patients with cerebral oligometastases depends to a great extent on the number and location of the metastases. While whole-brain radiotherapy (WBRT) is performed for diffuse metastasis, for oligometastases, stereotactic radiosurgery (SRS) is used to protect neurocognitive function [1–4]. In order to detect even the smallest BM, the diagnostic accuracy of the MR examination technique is thus critical.

In 2000, Hochstenbag et al. reported on the significance of the MRI examination in pretherapeutic staging of BM [5]. Through the use of MRI alone, the number of newly detected BM in SCLC patients increased by 15%. In this study on a 0.5T MRI, only T1-weighted spin echo (SE) sequences with a relatively large slice thicknesses of 10 mm were used. However, pulsation artifacts of the vessels in the posterior fossa result in poor image quality in standard SE images. Metastases in the brainstem or cerebellum can thus be missed. With the introduction of contrast-enhanced 3D GE T1-weighted MPRAGE (magnetization-prepared rapid gradient echo) images, Wenz et al. were able to detect considerably more metastases in 1.5T MRI than with SE sequences alone [6]. This was primarily due to the thinner slices. The superior spatial resolution of the 3D GE sequences is associated with a greater susceptibility for artifacts and poorer contrast compared with 2D SE sequences [7, 8]. With the introduction of new isotropic 3D fast (turbo) spin echo sequences, it is today possible to detect very small metastatic lesions (< 5 mm) in an acceptable examination time using 3.0T MRI. These sequences feature very long echo trains, lower flip angles, and ultrashort echo spacing. Mugler et al. developed a 3D fast SE sequence with variable flip angle refocusing pulses [9]. That which is termed the SPACE sequence solved the previous problems of poor 3D resolution of gradient echo sequences using sampling perfection with application-optimized contrast by using different flip angle evolutions. Due to the isotropic resolution, the voxels generated are the same size in every direction, allowing reconstruction in all three planes. Various studies have reported on the diagnostic significance of the SPACE sequence [10–12]. By suppressing the signal in the cortical vessels, the contrast enhancement of very small cerebral metastases is much clearer in 3D SPACE than in 3D MPRAGE images. Kato et al. showed in an initial study with 16 patients with BM that 3D SPACE visualizes metastases significantly better than 3D MPRAGE due to the better contrast rate (CR) [10].

The clinical significance of this fast (turbo) spin echo technique as black blood imaging for radiotherapy has not yet been studied. This clinical study aims to investigate whether 3D SPACE can be integrated in preradiotherapy staging and radiation treatment planning. The objective of the study is to clarify how many more metastases are detected using 3D SPACE compared with 3D MPRAGE in radiation treatment planning and what effect the higher number of metastases has on the later radiation technique (SRS versus WBRT). A metastasis count of ≤ 4 BM was specified as the upper limit for SRS according to the guidelines from 2015 [13].

Several authors have reported that the lesion contrast of BM increases with delayed imaging time [14, 15]. For departments that do not have access to black blood imaging sequences such as 3D SPACE for radiation treatment planning, it should be tested whether a 3D MPRAGE late-phase contrast-enhanced image can detect the same number of metastases as 3D SPACE.

## Materials and methods

### Patients

All patients had an external diagnostic MRI examination and were referred to our CyberKnife (Accuray Inc. Sunnyvale, CA, USA) center for SRS with suspected BM. The study enrolled 214 consecutive patients with brain oligometastases (no more than 4 lesions in diagnostic MRI, no extracranial metastases) who had an MRI for radiation planning for SRS between May 2016 and February 2018 at the Department of Radiation Oncology and Radiation Therapy at Heidelberg University Hospital. All patients were older than 18 years and had no contraindication to an MRI examination. The MRI data of 15 patients could not be evaluated because of the absence of BM in MRI-based radiation treatment planning (MPRAGE and SPACE sequences; *n* = 7 patients after chemotherapy) or had incomplete MRI sequences due to poor general condition (*n* = 8 patients). The patient characteristics of the remaining 199 patients are summarized in Table 1.

**Table 1:** Patient characteristics

*All patients, no*	199
*Gender, no. (%)*	
Female	112 (56.3)
Male	87 (43.7)
*Age, years*	
Median (range)	62 (20–90)
IQR	17
*Immunomodulating therapy, no. (%)*	
No immunomodulating therapy	143 (71.9)
Immunomodulating therapy	56 (28.1)
*Primary tumor type, no. (%)*	
Lung	110 (55.3)
Melanoma	29 (14.6)
Breast	28 (14.1)
Renal cell carcinoma	13 (6.5)
Cancer of unknown primary (CUP)	5 (2.5)
Others	14 (7.0)
*Previous radiotherapy to the brain, no. (%)*	
No SRS/WBRT	154 (77.4)
SRS	26 (13.1)
WBRT	19 (9.5)

*IQR* interquartile range, *SRS* stereotactic radiosurgery, *WBRT* whole-brain radiotherapy

^a^Percentages may not add up to 100 because of rounding

### Magnetic resonance imaging

MRI was carried out on a 3T scanner (Magnetom SKYRA®, Siemens Medical Systems, Erlangen, Germany) using a transmit/receive quadrature four-channel head coil. All scans were carried out without immobilization devices for use in treatment planning. The axial images were conducted without tilting in order to ensure a later fusion with CT examinations for radiation treatment planning.

The same imaging protocol was used in all patients and was standardized for timing and sequence order: a three-dimensional high-resolution T1-weighted gradient echo sequence (repetition time 2000 ms, echo time 2.44 ms, slice thickness 0.9 mm) for MPRAGE was carried out 2 min after intravenous administration of 0.1 mmol/kg body weight of gadobutrol (Gadovist, Bayer Schering Pharma, Berlin, Germany) with an additional saline flush of 30 ml. The imaging parameters for the SPACE were repetition time 700 ms, echo time 12 ms, and slice thickness 1.0 mm. The post-gadolinium late-phase MPRAGE images were made 10 min after contrast agent administration.

The MRI scans were reviewed by a specialized radiologist (TW) and a specialized radiation oncologist (SR) with > 15 years extensive experience in cerebral MRI. All cerebral lesions were evaluated based on the criteria published by Kato et al. [10]. First, the normal structures with contrast enhancement such as cortical vessels were differentiated from suspicious lesions. Of the suspicious cerebral contrast-enhancing lesions, the lesions not suspicious for metastasis (such as venous anomalies) were differentiated from the lesions suspicious for metastasis. All lesions that were diagnosed as cerebral metastases were included in the subsequent radiation treatment planning. For the contrast-enhanced MR sequences, the MPRAGE images were first evaluated, then the SPACE images, and finally the post-gadolinium late-phase MPRAGE images. We measured the shorter diameter of each lesion in the axial plane of the MPRAGE and SPACE images. After this reviewing process, an expert panel of radiation oncologists considered the scans again. The radiation therapy modality (SRS versus WBRT) was selected at the discretion of the treating radiation oncologists, based on the number of lesions, lesion size, location of the lesions, and the patient’s performance status.

The study protocol was approved by the local ethics committee. The study complies with the Helsinki Declaration, the American Medical Association’s professional code of conduct, the principles of Good Clinical Practice guidelines, and the Federal Data Protection Act.

### Statistical methods

The paired-samples *t*-test was used to compare the mean number of BM on the standard MPRAGE sequence with the mean number of metastases on the late-phase MPRAGE and SPACE sequences. Spearman rank-order correlation coefficients (r_s_) were computed to test for associations between the number of metastatic lesions on the early-phase MPRAGE sequence and the number of additional metastatic lesions on the late-phase MPRAGE and SPACE sequences. All calculations were performed using SPSS version 27 (IBM, Armonk, NY, USA) with *p* < 0.05 considered significant.

## Results

**Fig. 1:**
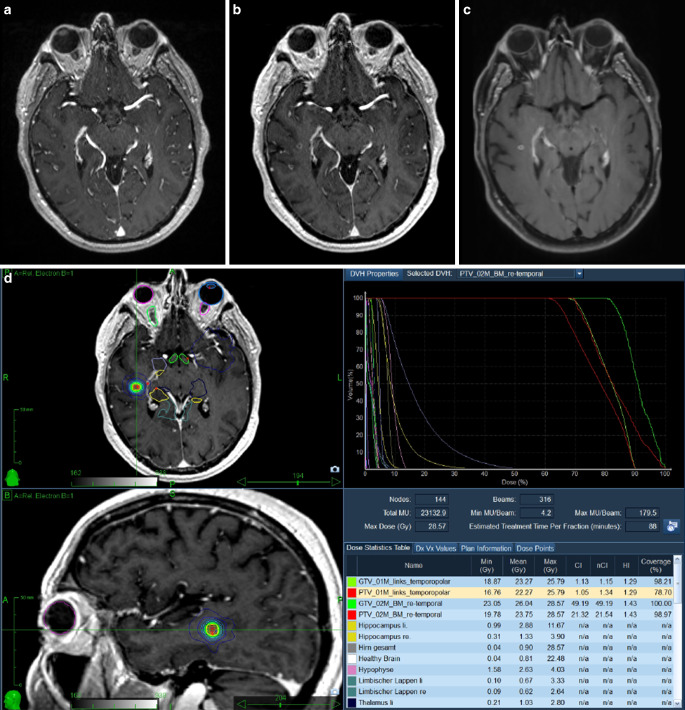
**a** MPRAGE post gadolinium: cystic metastasis in the right temporal lobe poorly differentiable from surrounding structures. **b** MPRAGE late post gadolinium: increasing contrast enhancement of the right temporal metastasis. **c** SPACE post gadolinium: metastasis clearly more differentiable from the cortical veins. **d** CyberKnife (Accuray Inc. Sunnyvale, CA, USA) treatment plan

**Fig. 2:**
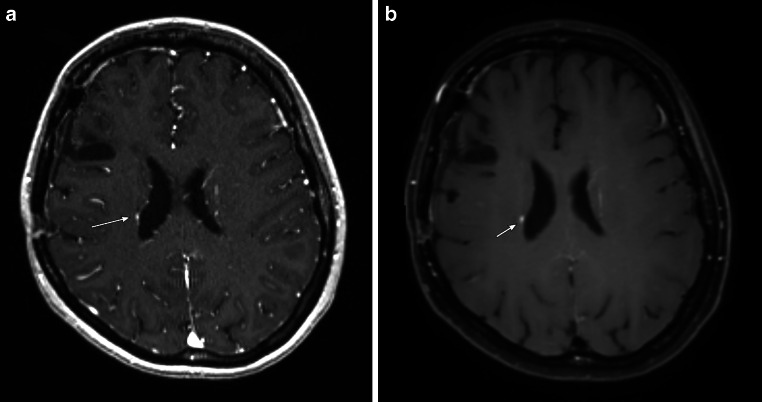
**a** MPRAGE post gadolinium: periventricular metastasis (*arrow*) poorly differentiable from periventricular vessels. **b** SPACE post gadolinium: significantly improved visualization of periventricular metastasis (*arrow*) due to signal suppression of periventricular vessels

A summary of the BM characteristics is shown in Table 2. Overall, we detected 29.8% more BM on the 3D SPACE sequences and 16.9% more BM on the 3D MPRAGE late-phase sequences than on the established standard 3D MPRAGE sequences. Incidental findings were detected in 3% of the 199 patients and included one meningioma and five developmental venous anomalies (DVA). The mean numbers of BM on the 3D SPACE and 3D MPRAGE late-phase sequences were significantly higher than those found on the standard 3D MPRAGE sequences (*p* < 0.0001, paired-samples *t*-test for both comparisons). Of note, lesions of less than 2 mm in diameter on the 3D SPACE sequence were not seen in either the established standard 3D MPRAGE or the 3D MPRAGE late-phase sequence. Metastatic lesions near the cortical vessels (Fig. 1c) and periventricular metastases (Fig. 2b) were particularly well detected by the 3D SPACE sequence.

**Table 2:** Brain metastases’ characteristics

	Early-phase MPRAGE	Late-phase MPRAGE	SPACE
*Total number*	681	796	884
*Mean (SD)*	3.4 (4.2)	4.0 (5.3)	4.4 (6.0)
*Median (range)*	2 (0–31)	2 (1–37)	2 (1–37)
*IQR*	3	3	4
*Grouping of patients by number of brain metastases, no. (%)*			
0	3 (1.5)	0	0
1–4	156 (78.4)	155 (77.9)	147 (73.9)
5–10	27 (13.6)	29 (14.6)	36 (18.1)
> 10	13 (6.5)	15 (7.5)	16 (8.0)

*IQR* interquartile range, *SD *standard deviation, *MPRAGE* magnetization-prepared rapid acquisition of gradient echo, *SPACE *sampling perfection with application-optimized contrast by using different flip angle evolutions

**Fig. 3:**
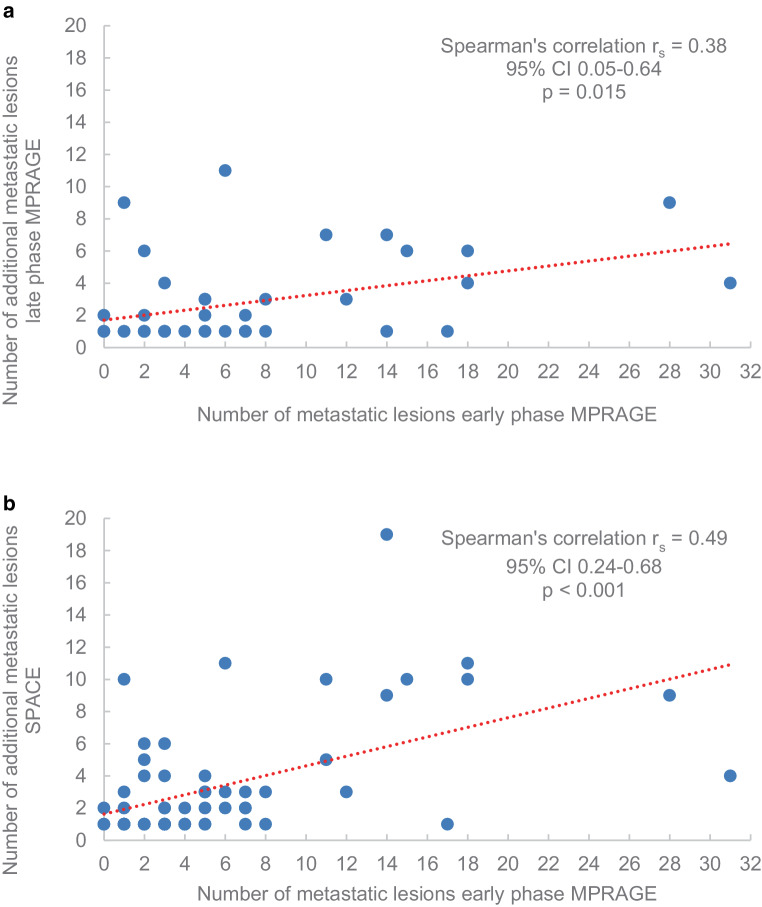
Correlation between the number of metastatic lesions in the early-phase MPRAGE sequence and the number of additional metastatic lesions in the late-phase MPRAGE sequence (**a**) and the SPACE sequence (**b**)

At the patient level, the 3D SPACE sequence showed 57.8% more metastatic lesions than the standard 3D MPRAGE sequence in 60 of the 199 patients (554 versus 351 metastases in 30.2% of the patients), and the 3D MPRAGE late-phase sequence detected 39.1% more lesions than the standard 3D MPRAGE sequence in 41 of the 199 patients (409 versus 294 metastases in 20.6% of the patients). The higher the number of BM detected in the standard 3D MPRAGE sequence, the higher the number of additional metastases in the 3D MPRAGE late-phase sequence (for details see Fig. 3).

### Modification of radiotherapy based on SPACE findings

In this study, 80% of the patients (*n* = 159) had cerebral oligometastases with between 0 and 4 BM. In a total of 125 oligometastatic patients in whom the standard early-phase MPRAGE showed 0–4 metastases, no additional metastases were found in the late-phase MPRAGE and SPACE scans. The SPACE scan alone detected 19 additional metastases in 16 oligometastatic patients, so SRS treatment was indicated for 12 patients. In 11 patients, these additional metastases could be irradiated using SRS. For one patient, the radiation treatment planning MRI showed additional calvarial metastases, so WBRT was carried out.

In 18 oligometastatic patients, more metastases were found simultaneously in the late-phase MPRAGE (*n* = 36) and in the SPACE (*n* = 51) scans than in the standard early-phase MPRAGE scan. In this patient group, the SPACE scan showed more metastases than the late-phase MPRAGE in 10 patients, so these patients were given a recommendation for SRS from the tumor board. SRS was carried out in a total of 9 patients. One patient with only one brain metastasis could not be treated with SRS because he could not tolerate prolonged immobilization during treatment. He was given WBRT with boost for this metastasis (details are shown in Table 3). Summarizing the two oligometastatic groups, a total of 20 oligometastatic patients were given SRS for the additional metastases detected solely on the basis of SPACE.

**Table 3:** Numbers (and percentages) of patients, metastases, and SRS treatments by grouping the patients with 0–4 metastases in the early-phase MPRAGE sequence according to the presence or absence of additional metastases on the SPACE and late-phase MPRAGE sequences

	Patients	Metastases				Decision for SRS based on SPACE imaging		SRS performed	
Detection of metastatic lesions relative to early-phase MPRAGE imaging		Early-phase MPRAGE	Late-phase MPRAGE	SPACE	Number of additional detected metastases	Patients (%)	Metastases (%)	Patients (%)	Metastases (%)
Additional metastases on SPACE imaging	16	35	35	54	SPACE: +19 Late-phase MPRAGE: +0	12 (75.0)	33 (61.1)	11 (91.7)	27 (81.8)
Additional metastases on SPACE ± late-phase MPRAGE imaging	18	35	71	86	SPACE: +51 Late-phase MPRAGE: +36	10 (55.6)	28 (32.6)	9 (90.0)	23 (82.1)
No additional metastases on SPACE + late-phase MPRAGE imaging	125	230	230	230	SPACE: +0 Late-phase MPRAGE: +0	125 (100)	230 (100)	121 (96.8)	221 (96.1)

*MPRAGE* magnetization-prepared rapid acquisition of gradient echo, *SPACE *sampling perfection with application-optimized contrast by using different flip angle evolutions

## Discussion

Several studies have shown that black blood (BB) MRI (SPACE, VISTA) detects significantly more brain lesions < 5 mm [10, 16, 17]. This study showed for the first time that BB imaging (SPACE) can be effectively integrated into stereotactic radiation treatment planning with the CyberKnife. Among the multifactorial causes for the superior detectability of BM using SPACE are the better signal-to-noise ratio and the higher spatial resolution in the submillimeter range. The sequence is clearly superior to MPRAGE in detecting smaller short-axis diameters and thus also smaller metastases. However, false-positive findings due to insufficient suppression of cortical vessels or small contrast enhancement of an immunological inflammatory response due to antibody treatment can be misinterpreted to be a metastasis.

In the late-phase MPRAGE imaging, cerebral metastases show contrast blush as an expression of neovascularization compared with early-phase MPRAGE imaging (Fig. 1b). In addition, washout of the contrast agent was seen in the cerebral vessels in late-phase MPRAGE imaging (Fig. 1b). This facilitates better differentiation of the intracerebral vessels from metastases in late-phase MPRAGE imaging as well.

The 3D SPACE image showed more intracerebral metastases and, above all, smaller metastases in MRI-based radiation treatment planning compared with conventional 3D MPRAGE. These metastases would not have been included in radiation treatment if only contrast-enhanced 3D MPRAGE had been used. This would have led later to a poorer clinical outcome of the patients, as a total of 197 metastases were not detected in conventional 3D MPRAGE. Another advantage is the administration of one dose of MR contrast with a good signal-to-noise ratio of 3D SPACE even in the submillimeter range. The administration of three times as much gadolinium, as described by Donahue et al. when using 1‑mm thick MPRAGE sequences, is not necessary with 3D SPACE [18]. The good spatial resolution allowed false-positive lesions such as cortical veins to be recognized and excluded from the radiation treatment planning [10].

Image acquisition for radiation treatment planning places special demands on MRI. For radiation treatment planning for CyberKnife radiosurgery on the cranium in particular, slice thicknesses less than 1 mm are required with three-dimensional resolution and a good signal-to-noise ratio. This would not be possible with conventional 2D T1 spin echo sequences. In addition to good spatial resolution, low geometric distortion in the MR image is also important. The sharp delineation of the contrast enhancement of a metastasis in 3D SPACE allows precise determination of the target volume with a steep dose gradient, while simultaneously protecting the surrounding brain tissue. For the radiotherapist, detection and target volume contouring of small BM are considerably easier in 3D SPACE, as there is no interference from cortical veins.

Our patients were treated between 2016 and 2018, at a time when SRS of brain metastases was limited to up to four brain metastases. Now it is not uncommon to treat up to 10 metastases or more [19]. Therefore, the change in treatment concept based on SPACE imaging needs to be considered in the current management of BM, as many centers would still offer SRS for more brain lesions. We assume that the more lesions are included in the treatment concept, the greater the benefit of SPACE imaging.

One limitation of the study is that as in earlier studies, there is no histopathological confirmation of the irradiated brain lesion. Due to the poor prognosis and high mortality rate, not all patients were available for follow-up examinations.

Another limitation is that we used a 12-channel head coil for acquisition of the 3D SPACE and 3D MPRAGE sequences for radiation treatment planning. According to Reichert et al., we would have needed 30% less time if a 32-channel head coil had been used [20].

## Conclusion

Using 3D SPACE is a practical alternative to the routinely used T1-weighted 3D MPRAGE, especially for radiosurgery treatment planning for BM. Our data show that 3D SPACE allows detection of significantly more cerebral metastases that can be included in radiation treatment planning. For radiotherapists, the increasing use of SRS even for patients with up to 10 BM increases the importance of precise pretherapeutic detection of all cerebral lesions in the MRI examination for radiation treatment planning [19, 21]. The further clinical significance of using 3D SPACE for radiation treatment planning on patient outcome and quality of life after radiosurgery is currently being tested in an ongoing two-armed prospective randomized phase II study at our center (Clinical Trial Code: NCT03303365).

## Abbreviations

BM: Brain metastasis
CK: CyberKnife
CR: Contrast rate
GE: Gradient echo
MPRAGE: Magnetization-prepared rapid acquisition of gradient echo
MRI: Magnetic resonance imaging
SE: Spin echo
SPACE: Sampling perfection with application-optimized contrast by using different flip angle evolutions
SRS: Stereotactic radiosurgery
WBRT: Whole-brain radiotherapy

## References

[1] Robin TP, Rusthoven CG (2018). Strategies to preserve cognition in patients with brain metastases: a review. Front Oncol.

[2] Clarke JWRS, McGregor JM, Grecula JC, Mayr NA, Wang JZ, Li K, Grupta N, Kendra KL, Olencki TE, Cavaliere R, Sarkar A, Lo SS (2010). Stereotactic radiosurgery with or without whole brain radiotherapy for patients with a single radioresistent brain metastasis. Am J Clin Oncol.

[3] Shen CJ, Kummerlowe MN, Redmond KJ, Rigamonti D, Lim MK, Kleinberg LR (2016). Stereotactic radiosurgery: treatment of brain metastasis without interruption of systemic therapy. Int J Radiat Oncol Biol Phys.

[4] Pazzaglia S, Briganti G, Mancuso M, Saran A (2020). Neurocognitive decline following radiotherapy: mechanisms and therapeutic implications. Cancers.

[5] Hochstenbag MM, Twijnstra A, Wilmink JT, Wouters EF, ten Velde GP (2000). Asymptomatic brain metastases (BM) in small cell lung cancer (SCLC): MR-imaging is useful at initial diagnosis. J Neurooncol.

[6] Wenz F, Hess T, Knopp MV, Weisser G, Blüml S, Schad LR, Hawighorst H, van Kaick G (1994). 3D MPRAGE evaluation of lesions in the posterior cranial fossa. Magn Reson Imaging.

[7] Brant-Zawadzki M, Gillan GD, Nitz WR (1992). MP RAGE: a three-dimensional, T1-weighted, gradient-echo sequence—initial experience in the brain. Radiology.

[8] Kakeda S, Korogi Y, Hiai Y, Ohnari N, Moriya J, Kamada K, Hanamiya M, Sato T, Kitajima M (2007). Detection of brain metastasis at 3T: comparison among SE, IR-FSE and 3D-GRE sequences. Eur Radiol.

[9] Mugler JP, Bao S, Mulkern RV, Guttmann CR, Robertson RL, Jolesz FA, Brookeman JR (2000). Optimized single-slab three-dimensional spin-echo MR imaging of the brain. Radiology.

[10] Kato Y, Higano S, Tamura H, Mugikura S, Umetsu A, Murata T, Takahashi S (2009). Usefulness of contrast-enhanced T1-weighted sampling perfection with application-optimized contrasts by using different flip angle evolutions in detection of small brain metastasis at 3T MR imaging: comparison with magnetization-prepared rapid acquisition of gradient echo imaging. AJNR Am J Neuroradiol.

[11] Kwak HS, Hwang S, Chung GH, Song JS, Choi EJ (2015). Detection of small brain metastases at 3 T: comparing the diagnostic performances of contrast-enhanced T1-weighted SPACE, MPRAGE, and 2D FLASH imaging. Clin Imaging.

[12] Komada T, Naganawa S, Ogawa H, Matsushima M, Kubota S, Kawai H, Fukatsu H, Ikeda M, Kawamura M, Sakurai Y (2008). Contrast-enhanced MR imaging of metastatic brain tumor at 3 tesla: utility of T(1)-weighted SPACE compared with 2D spin echo and 3D gradient echo sequence. Magn Reson Med Sci.

[13] Weller M (2015). Leitlinien für Diagnostik und Therapie in der Neurologie: Hirnmetastasen und Meningeosis neoplastica.

[14] Yuh WT, Tali ET, Nguyen HD, Simonson TM, Mayr NA, Fisher DJ (1995). The effect of contrast dose, imaging time, and lesion size in the MR detection of intracerebral metastasis. AJNR Am J Neuroradiol.

[15] Kushnirsky MNV, Katz JS, Steinklein J, Rosen L, Warshall C, Schulder M, Knisely JP (2016). Time-delayed contrast-enhanced MRI improves detection of brain metastases and apparent treatment volumes. J Neurosurg.

[16] Yoneyama M, Obara M, Takahara T, Kikuchi K, Nakamura M, Tatsuno S, Sawano S, Ogino T, Togao O, Yoshiura T (2014). Volume isotropic simultaneous interleaved black- and bright-blood imaging: a novel sequence for contrast-enhanced screening of brain metastasis. Magn Reson Med Sci.

[17] Kaufmann TJ, Smits M, Boxerman J, Huang R, Barboriak DP, Weller M, Chung C, Tsien C, Brown PD, Shankar L (2020). Consensus recommendations for a standardized brain tumor imaging protocol for clinical trials in brain metastases. Neuro Oncol.

[18] Donahue BR, Goldberg JD, Golfinos JG, Knopp EA, Comiskey J, Rush SC, Han K, Mukhi V, Cooper JS (2003). Importance of MR technique for stereotactic radiosurgery. Neuro Oncol.

[19] Yamamoto M, Serizawa T, Shuto T, Akabane A, Higuchi Y, Kawagishi J, Yamanaka K, Sato Y, Jokura H, Yomo S (2014). Stereotactic radiosurgery for patients with multiple brain metastases (JLGK0901): a multi-institutional prospective observational study. Lancet Oncol.

[20] Reichert M, Morelli JN, Runge VM, Tao A, von Ritschl R, von Ritschl A, Padua A, Dix JE, Marra MJ, Schoenberg SO (2013). Contrast-enhanced 3-dimensional SPACE versus MP-RAGE for the detection of brain metastases: considerations with a 32-channel head coil. Invest Radiol.

[21] Acker G, Hashemi S-M, Fuellhase J, Kluge A, Conti A, Kufeld M, Kreimeier A, Loebel F, Kord M, Sladek D (2020). Efficacy and safety of CyberKnife radiosurgery in elderly patients with brain metastases: a retrospective clinical evaluation. Radiat Oncol.

